# Complementary and Alternative Therapies for Autism Spectrum Disorder

**DOI:** 10.1155/2015/258589

**Published:** 2015-05-07

**Authors:** Natascia Brondino, Laura Fusar-Poli, Matteo Rocchetti, Umberto Provenzani, Francesco Barale, Pierluigi Politi

**Affiliations:** Department of Brain and Behavioral Sciences, Section of Psychiatry, University of Pavia, Via Bassi 21, 27100 Pavia, Italy

## Abstract

*Background*. Complementary and alternative medicine (CAM) represents a popular therapeutic option for patients with autism spectrum disorder (ASD). Unfortunately, there is a paucity of data regarding the efficacy of CAM in ASD. The aim of the present systematic review is to investigate trials of CAM in ASD. *Material and Methods*. We searched the following databases: MEDLINE, EMBASE, Cochrane Database of Systematic Reviews, CINAHL, Psychology and Behavioral Sciences Collection, Agricola, and Food Science Source. *Results*. Our literature search identified 2687 clinical publications. After the title/abstract screening, 139 publications were obtained for detailed evaluation. After detailed evaluation 67 studies were included, from hand search of references we retrieved 13 additional studies for a total of 80. *Conclusion*. There is no conclusive evidence supporting the efficacy of CAM therapies in ASD. Promising results are reported for music therapy, sensory integration therapy, acupuncture, and massage.

## 1. Introduction

Autism spectrum disorder (ASD) is a heterogeneous group of neurodevelopmental conditions characterized by the presence of impaired social communication and reciprocity and of restricted and stereotyped pattern of behaviors and interests. In the last few decades, the prevalence of ASD has increased dramatically, configuring a sort of “epidemics” [1]. Usually, it affects boys more than girls (4 : 1 ratio) and it is a lifelong condition, generally diagnosed in early childhood [2]. Despite the public concern about this phenomenon and the interest of the scientific community, there are still controversies about ASD etiology. It is hypothesized that ASD is caused by a combination of genetic and environmental stimuli, but no clear pathogenesis has been defined [3]. Effective therapies for ASD core symptoms have not yet been developed. Evidence-based first-line treatments are represented by behavioral therapies (such as TEACCH or ABA) [4]. Use of pharmacological medications (such as risperidone or aripiprazole) [5, 6] is usually limited to treating behavioral symptoms of the disorder like irritability or aggressiveness. Unfortunately, despite the dramatic effect sizes for these problem behaviors, the pharmacological approach to core symptoms has led to inconclusive results and is sometimes burdened by severe adverse events [7]. Families of children with autism are usually worried by potential drug side effects and are continuously looking for treatments which are more secure. As a consequence, in recent years, there has been an increasing interest for complementary and alternative medicine, not only in ASD, but also in several pathological conditions.

According to the definition of the Cochrane Collaboration, “complementary and alternative medicine (CAM) is a broad domain of healing resources that encompasses all health systems, modalities, and practices and their accompanying theories and beliefs, other than those intrinsic to the politically dominant health system of a particular society or culture in a given historical period. CAM includes all such practices and ideas self-defined by their users as preventing or treating illness or promoting health and well-being. Boundaries within CAM and between the CAM domain and that of the dominant system are not always sharp or fixed.” CAMs have become very popular therapies among adults and children with ASD [8]. In 2006, according to an Internet survey involving a sample of 540 families from the Autism Society of America and the autism organizations worldwide, each family with a child with ASD has tried a mean of 7 treatments [9], among which many were CAMs. Particularly, it has recently been estimated that 28% of children with ASD are treated with CAM [8]: CAM usage appears to be more common among Caucasian families with high economic income. It is of note that even before the diagnosis, nearly one-third of children have already received a CAM treatment and 9% of this population has used a potentially harmful therapy such as chelation [10]. Previously, higher CAM usage among families with an ASD child had been reported (ranging from 52 to 74%) [11, 12]. Compared to the work of Perrin et al., previous studies considered a wider range of CAMs and enrolled older subjects as participants, which could explain the resulting higher figures. Type of CAM use appears to be stable over time: biological therapies, in particular elimination or special diets, are the most frequent [8–12].

Despite its popularity, the use of CAMs in ASD is controversial; recently more methodologically sounded trials have been designed and conducted in order to test the efficacy of different CAM therapies, and the knowledge about CAMs is moving from an anecdotal form to a more scientific one. The aim of the present systematic review is to give a comprehensive overlook of the efficacy of CAM in ASD.

## 2. Material and Methods

In October 2014, we searched the following databases: MEDLINE, EMBASE, Cochrane Database of Systematic Reviews, CINAHL, Psychology and Behavioral Sciences Collection, Agricola, and Food Science Source. The search terms were as follows: ‘asd'/exp OR asd OR ‘autism'/exp OR autism AND (‘spectrum'/exp OR spectrum) AND (‘disorder'/exp OR disorder) OR autistic^*^ AND complementary OR alternative AND (‘medicine'/exp OR medicine) OR herbal OR ‘music'/exp OR music AND (‘therapy'/exp OR therapy) OR ‘dance'/exp OR dance AND (‘therapy'/exp OR therapy) OR ‘diet'/exp OR diet OR cam OR ‘yoga'/exp OR yoga OR supplement OR plant OR botanical. The search strategy had no time restriction but was limited to articles in English. Additionally, all recovered papers were reviewed for further relevant references. Researchers in the field were reached to obtain additional or unpublished data, if available.

Two researchers (Natascia Brondino and Laura Fusar-Poli) independently reviewed all information about the articles provided by the databases. Any discrepancies were solved by consensus.

Our inclusion criteria were broad on study design, including both randomized and open label trials, yielding primary results on the effects of CAM administration in core symptoms of ASD. ASD was defined according to internationally valid diagnostic criteria such as the International Classification of Diseases (ICD) or the Diagnostic and Statistical Manual of Mental Disorders (DSM). We excluded case report and case series. We did not consider off-label drugs (such as oxytocin, secretin, and antibiotics) as CAM. Additionally, we did not include trials on melatonin in ASD (for this purpose see the review written by Tordjman et al. [93] in 2013) as it is generally used to treat sleep problems in autistic patients and not intended to treat core symptoms of ASD.

## 3. Results

Our literature search identified 2687 clinical publications. After the title/abstract screening, 139 publications were obtained for detailed evaluation. After detailed evaluation 67 studies were included; from hand search of references we retrieved 13 additional studies for a total of 80.

## 4. Biologically Based Treatments

Biological CAM treatments usually include dietary interventions, vitamin supplements, and herbal remedies [8]. There are several critical mechanisms that could be advocate to explain the biological effect of CAM: in particular, researchers in the field have pointed out how Natural Killer (NK) cell activation or immune system modulation may play a key role in several biological CAMs (i.e., elimination diets or probiotics) [94]. Additionally, other potential pathways involved in biologically based CAMs include antioxidant and anti-inflammatory activity (i.e., flavonoids and probiotics) [95], neuroprotection (i.e., omega 3) [96], or modulation of the neurotransmitter-induced response (i.e., L-carnosine) [97]. In addition, more discussed CAMs (Hyperbaric Oxygen Therapy (HBOT) or chelation) could be included in this section as the rationale for their use relies on a biological mechanism. In particular, chelation tries to eliminate toxic metals from blood [98] while HBOT aims at enhancing blood oxygen level in order to determine a positive impact on several neurological functions such as language, memory, and cognition [99].

### 4.1. Dietary Interventions

Among CAMs currently used in autism, elimination diets, especially gluten and/or casein-free diets, are one of the most popular (Table 1). In fact, Levy and Hyman have reported that 1 in every 7 children is put on this nutritional regimen [100]. The rationale behind the use of a specific dietary regimen relies on the presence of specific food allergen (such as casein or gluten) which could enhance immune response in predisposed subjects or trigger autoimmunity [101, 102]. Another theory is that gluten and casein may originate opiate-active metabolites in the gut that could reach the systemic circulation (the “opioid excess theory” of autism) [103]. Additionally, several gastrointestinal abnormalities have been observed in subjects with ASD, such as increased permeability of the gut barrier and bacterial overgrowth which could benefit from elimination diet [34, 104]. Elimination diets showed modest clinical effect in treating children with Attention-Deficit Hyperactivity Disorder (ADHD) [105], which shares some features with ASD [106].

Focusing on autism, the first studies investigating the efficacy of a gluten- and casein-free diet were conducted in the 1990s and were mostly uncontrolled trials (i.e., [107–109]). Although all these reports showed a significant improvement of ASD symptoms after the elimination diet, there were several methodological flaws, such as the lack of a control group, poor diagnostic characterization, small sample sizes, use of unstandardized outcome measures, and absence of control on dietary adhesion. The first randomized controlled trial was done in 2002 [13]: the authors enrolled 10 pairs of autistic children matched for age, cognitive level, and severity. In each pair, one child was randomly assigned to a gluten- and casein-free (GFCF) diet while the other maintained the normal diet. The follow-up time was one year. It was a single blind study and the authors observed modification of attention, social and emotional factor, cognitive level, language, and motor skills in the elimination diet group. Unfortunately, the study is flawed by several caveats such as the inclusion of patients with “abnormal urinary peptide patterns,” which could limit the generalizability of the findings, the single blind design, and the lack of dietary fidelity evaluation and of internationally valid outcome measures. Later on, Elder et al. [14] conducted a randomized, double blind, repeated-measure, crossover trial evaluating the efficacy of GFCF diet in 15 children with ASD. Patients were on diet for 12 weeks. Group data indicated no statistically significant findings. In 2010, Whiteley et al. [15] enrolled 72 children with ASD who were randomly assigned to the GFCF diet or a control group. The overall attrition rate was high (11%): at the 12-month follow-up only 28 children remained in the GFCF group and 29 in the normal diet. At the same time-point, while patients in the GFCF continued their diet until the end of the study, children eating a normal diet were switched to the GFCF (however, only if their outcome measures at 12 months exceeded a predefined, but unclear, threshold). At the 24-month follow-up only 18 children did not drop out from the GFCF diet. The authors assessed ASD symptoms at baseline and 8, 12, 20, and 24 months, through the Autism Diagnostic Observation Schedule-Generic (ADOS-G), the Vineland Adaptive Behavior Scale (VABS), and Gilliam Autism Rating Scale (GARS). It is unclear on what patient groups or time-points the statistical analysis was carried out. The authors reported a significant improvement in social symptoms (measured only with GARS) and communication and repetitive behavior (ADOS assessed). However, the observed differences did not seem clinically meaningful. In 2011, Johnson et al. [16] piloted a three-month, prospective, randomized, parallel group trial. Twenty-two children with ASD were randomized to the GFCF diet or a healthy, low sugar diet. No statistically significant differences in core symptoms were reported between GFCF diet and control diet. The GFCF diet did not determine more side effects than the healthy diet. Unfortunately, adherence to the GFCF diet proved to be difficult.

Among other less common dietary interventions for autism, the ketogenic diet should be mentioned. The ketogenic diet is low-carbohydrate, high-fat diet which has been successfully administered in children with refractory epilepsy: this dietary regimen determines a better seizure control and has an effect comparable to antiepileptic drugs [110]. Evangeliou et al. (2003) [17] conducted a prospective follow-up trial evaluating the use of the ketogenic diet in 30 children with ASD. The diet was based on the John Radcliffe diet, which consisted of the following regimen: 30% of energy derived from medium-chain triglyceride oil, 30% from fresh cream, 11% from saturated fat, 19% from carbohydrates, and 10% from protein. This dietary treatment appeared more easy to follow and manage than the proper ketogenic diet. The diet was administered for 4 weeks, followed by 2 weeks of normal nutritional regimen. This cycle was repeated for 6 months. Overall, twelve patients discontinued the diet. The subjects who completed the study reported at least a minor improvement in CARS scores. However, this study suffers from an unblinded design, a high drop-out rate, and a poor diagnostic characterization of the participants.

IgE and non-IgE-mediated food allergies are highly prevalent among children. Food allergy could determine the onset of different neuropsychiatric symptoms, such as hyperactivity, or worsen behaviors already present in young patients with ASD [111]. Moving from this hypothesis, Karkelis et al. (2010) [18] tested a new diet in which children were randomly assigned to an elemental formula (containing free amino acids-Neocate) diet with exclusion of all milk products or to their previous normal diet. Study participants were also subdivided according to the presence or absence of IgE for cow milk. After 4 months, the authors reported a significant reduction of hyperactivity.

Traditional Chinese Medicine (TCM) has been practiced in Eastern countries for over 2000 years. It is based on a unique theoretical approach to diagnosis and treatment. It works on balancing opposing energies (yin and yang) and the life force (qi), which are present in everybody. According to the National Center for Complementary and Alternative Medicine, TCM comprehends several practices “including acupuncture, moxibustion (burning an herb above the skin to apply heat to acupuncture points), Chinese herbal medicine, tui na (Chinese therapeutic massage), dietary therapy, and tai chi and qi gong” [112]. Among TCM diets, the Chanyi approach suggests to decrease the intake of some foods (like meat and fish, eggs, ginger, garlic, and onion) which are thought to produce higher internal heat and exert a negative impact on the child's mood and cognitive functions. Chan et al. [19] conducted a double blind randomized study in which 24 ASD children were assigned either to a specific diet modification based on the Chanyi approach or to their usual diet for one month. The authors observed a significant improvement in parent-rated social problems and repetitive behaviors.

To date, the only functional food tested in autism is camel milk. Camel milk contains less cholesterol and lactose than cow milk and more vitamins and enzymes such as the peptidoglycan recognition protein (PGRP), which plays a role in preventing food allergy and modulating the immune system [20]. Two placebo controlled double blind randomized trials [20, 21] showed significant improvement in CARS scores and in antioxidant activity in children treated either with raw or boiled camel milk for 2 weeks compared to placebo.

### 4.2. Nutraceuticals

The term nutraceutical is defined as “any substance that is food or a part of food and provides medical or health benefits, including the prevention and treatment of disease” [113]. Usually, nutraceuticals consist of dietary supplements (such as vitamins, minerals, amino acids, and herbal substances) or functional food. Nutraceuticals could represent a potential treatment for autism with limited or no side effects, and they are commonly used in families with ASD (Table 2) [8].

#### 4.2.1. Omega 3

Among nutraceuticals, one of the most popular is omega 3 supplementation. Omega 3 fatty acids are essential polyunsaturated fatty acids, derived mainly from fish and seafood (the eicosapentaenoic acid (EPA) and the docosahexaenoic acid (DHA)) or seeds and grains (the alpha-linolenic acid (ALA)). The hypothesis behind the use of omega 3 in autism is still not completely formulated: it is, however, well known that omega 3 fatty acids are essential for brain development and function [114]. A recent Cochrane review [115] has meta-analyzed the findings of two randomized trials (total sample size = 37) [22, 23], evaluating the effect of omega 3 supplementation compared to placebo in children with ASD. Omega 3 dosage varied from 1.3 g/day to 1.5 g/day. Supplementation lasted for six weeks [22] and 12 weeks [23], respectively. Both studies used at least one common outcome measure, the Aberrant Behavioral Checklist (ABC). Overall, there was no significant effect of omega 3 supplementation on social interaction, communication, stereotypy, or hyperactivity. From this meta-analysis only two double blind placebo controlled trials were published. In one study [24], the authors recruited 57 children with ASD who were assigned to 1.3 g/daily of omega 3 or placebo for six weeks. Parent-rated symptoms were evaluated through an Internet questionnaire each week. Study findings showed no significant difference between omega 3 supplementation and placebo. Recently, 48 children with ASD [27] were randomized in a double blind fashion to receive DHA (200 mg/daily) or a placebo for 6 months. Outcome measures were the Clinical Global Impressions-Improvement scale, the Child Development Inventory, the ABC, and the Behavior Assessment Scale for Children. No significant difference was reported between the two groups. Two nonrandomized studies [25, 26] showed contrasting results: however, several methodological flaws were present in each study and also type and dosage of omega 3 supplementation varied greatly between the trials.

#### 4.2.2. Vitamins

Vitamin supplementation is another popular CAM therapy in ASD. The rationale for this treatment is based on the frequently observed dietary deficiency of vitamins and micronutrients in children with ASD. In fact, it has been reported that children with ASD introduce less than recommended amounts of calcium, vitamin D, vitamin K [116], vitamin A, vitamin E [117], zinc, vitamin B6 [118], and tetrahydrobiopterin [119]. These deficiencies could be the result of food selectivity or altered gastrointestinal absorption [118]. Several trials evaluating vitamins supplementation in ASD have been conducted. A recent Cochrane systematic review [120] evaluated the efficacy of combined vitamin B6-magnesium supplementation in ASD. The use of vitamin B6 moved from early data (1968) showing language improvement in autistic children treated with this nutraceutical. The combination of vitamin B6 with magnesium was postulated as magnesium could counteract several side effects connected with B6 supplementation (such as enuresis and irritability) [120]. Three randomized controlled studies were included [28–30], but data could not be meta-analyzed due to substantial heterogeneity between the trials. It is of note that all three trials reported no statistically significant difference between vitamin B6 supplementation and placebo. However, results could not be regarded as conclusive because several limitations in the study design should be taken into consideration such as small sample sizes and flawed data reporting.

Vitamin B12 was tested in two trials: the first from Bertoglio et al. [31] was a double blind, placebo controlled, randomized, crossover trial evaluating methyl B12 alone in children with ASD. The authors did not observe any significant difference between active treatment and placebo. However, in a post hoc analysis they were able to identify a subgroup of patients (30%) which could be rated as clinically improved after the active treatment but not after the placebo. Subsequently, Frye et al. [32] conducted an open label trial in which children were administered methyl B12 and folinic acid for three months. The authors reported a significant improvement in Vineland total and subscales score. However, given the study design, no precise conclusion could be drawn from the findings; additionally the authors included only patients with abnormal redox metabolism which could in turn limit the generalizability of the results to the entire ASD population. Furthermore, methyl B12 is administered through injection, a procedure which could be uncomfortable for children and adults with ASD: this fact could potentially explain the high drop-out rate (almost 25%).

Vitamin C supplementation alone is not so common in ASD. However, Dolske et al. [33] observed a reduction in stereotyped behaviors in a 30-week, double blind, placebo controlled trial in 18 children with ASD.

Multivitamin supplements were tested in a double blind randomized trial [121]. A common commercial vitamin supplement (containing several vitamins, minerals, no copper and iron, and antioxidants such as coenzyme Q10 and n-acetyl cysteine) was chosen as active treatment. The authors recruited 141 children with ASD who were randomly assigned to active treatment or placebo. The dosage was adjusted according to the child's weight and titrated to the full dose in three weeks. Supplementation lasted for three months. The authors observed improvement in parent-rated scores of irritability. However, the study suffered from a poor characterization of participants, which could be on psychotropic medications and put on different elimination diets. Additionally, the use of a multivitamin supplement prevented identifying single contributions of different vitamins and minerals.

Tetrahydrobiopterin (BH4), as other vitamins and micronutrients, is a natural substance that plays an essential role in several biochemical pathways. It has been tested as a therapeutic treatment in ASD in three trials. The first from Danfors et al. [35] was a randomized, double blind, placebo controlled, crossover study. Children with ASD were randomized to either BH4 or placebo for three months and then switched for other three months. No significant change was detected; only post hoc analysis revealed minor changes in secondary outcome measures. On the other hand, more recently, an open label trial [36] was aimed at testing BH4 in children with ASD and low cerebrospinal fluid level of BH4. The authors observed significant changes in several outcome measures on language production, social communication, activity of daily living, and irritability. However, the sample was very small and BH4 dosage was very high compared to previous reports [35]: it is of note that only 20% of subjects reported adverse events such as insomnia, irritability, and mild stomach discomfort. Another recent double blind, randomized, placebo controlled study [37], using a protocol similar to a previous study [35], did not report improvement in the primary outcome measure (Clinical Global Impression-Improvement and Severity): however, post hoc analysis on secondary outcome measures showed improvement in the BH4 group in social awareness and reduction of hyperactivity, mannerisms, and inappropriate speech.

#### 4.2.3. L-Carnosine

L-Carnosine is another CAM therapy tested in autism. The rationale for the use of this nutraceutical relies on the connection between carnosine and GABA functioning, which seems to be altered in ASD [122]. In particular carnosine could alter neurotransmission by interacting with zinc and copper at GABA receptor level [123]. In the only double blind, placebo controlled, randomized study [38] conducted so far, 400 mg of L-carnosine were administered twice daily to 17 children with ASD, while 14 children received the placebo. The follow-up lasted for 8 weeks. The authors found that supplementation with carnosine improved receptive speech and social behavior, with no side effect (apart from rare hyperactivity which disappeared after lowering the dose). It is of note that change in Clinical Global Impression rating did not reach significance, thus reducing the validity of the results.

#### 4.2.4. Flavonoids

The presence of altered redox status and concomitant subclinical inflammation has been reported in ASD [124]. Natural flavonoids, in particular quercetin and luteolin, exert a powerful antioxidant activity and have a low redox potential which could in turn be useful in autism [125]. Unfortunately, only one open label prospective trial [39] has evaluated a formula containing luteolin (100 mg/capsule, from chamomile), quercetin (70 mg/capsule), and the quercetin glycoside rutin (30 mg/capsule) in 50 children with ASD. Only 40 subjects completed the 26-week follow-up. Significant changes in adaptive functioning and aberrant behaviors were observed. The most relevant adverse event was irritability, which was experienced by half of the sample usually at the beginning of therapy (1–8 weeks).

#### 4.2.5. Probiotics

According to the World Health Organization, probiotics are live microorganisms which could exert health benefits on the host. Generally, probiotics are bacteria which belong to two groups,* Lactobacillus* or* Bifidobacterium* spp. In recent years, the gut-brain connection in autism has obtained much relevance: in fact, it is well known that gastrointestinal tract and brain can influence each other. Particularly, gut inflammation or altered microflora could determine a detrimental effect on brain development and function [126]. Moving from these premises, in 2012, 22 children with ASD were recruited in an open label trial [40]. They received two capsules daily of* Lactobacillus acidophilus* for two months. The authors observed significant modification in urinary excretion of arabinol and, concomitantly, significant improvement in core symptoms of autism, such as eye contact and correct recognition of human emotion.

#### 4.2.6. Digestive Enzymes

Moving from the hypothesis of gut abnormalities in autism, a double blind, placebo controlled, randomized, crossover trial has evaluated supplementation with digestive enzymes in autism [41]. The supplement was composed of three plant-derived enzymes (peptidase, protease 4.5, and papain) and was administered for three months; the active phase was preceded/followed by a placebo phase of the same duration. Of the 43 children enrolled, 16 did not complete the trial. Overall, there was no significant clinical change in autistic symptoms between enzyme treatment and placebo. It is of note that the authors observed that patients on the active treatment displayed higher food variety.

#### 4.2.7. Herbal Remedies

Among herbal remedies, a recent study from Chan et al. [44] investigated the potential usefulness of intranasally administered Borneol and Borax (two herbs which in Chinese traditional medicine were thought to enhance cognitive abilities) in 15 children with ASD. They recruited additionally 15 children with ASD which acted as a control group. This pilot study lasted for six months. The authors reported that subjects in the experimental group showed more flexibility in problem solving, greater attention, and planning capacities. Yokukansan, a traditional Japanese herbal remedy used for restlessness and behavioral symptoms of dementia, was tested in a 12-week, open label trial [43] in which the herb was administered to 40 subjects with Asperger syndrome or PDD-NOS. The dose was gradually titrated from 2.5 g/day to a maximum of 7.5 g/day. 90% of the sample showed a clinically significant response, and no serious adverse event was reported (only mild nausea in five patients).* Ginkgo biloba*, which could exert a useful anti-inflammatory activity and potentially enhance cognitive function [127], was evaluated in a study involving 47 children with autism [42]. Patients were randomly assigned to either* Ginkgo biloba* or placebo in adjunction to risperidone. The primary outcome was the ABC scale. There was no statistically significant difference between the two groups according to the aforementioned subscale. Thus* Ginkgo biloba* did not seem to be an efficacious adjunctive therapy to risperidone. However, it appeared to be safe and well tolerated even in childhood.

### 4.3. Hyperbaric Oxygen Therapy

Hyperbaric Oxygen Therapy (HBOT) is generally used to treat carbon monoxide poisoning or air embolism. The exact mechanism of action is not yet fully understood but HBOT seems to exert positive effects on different neurological symptoms [99, 128]. HBOT has been tested in four different trials with inconsistent results (Table 3). Particularly, two well-designed, randomized trials yielded opposite findings: Rossignol et al. [47] showed significant improvement while Granpeesheh et al. [45] observed no difference between the active and control conditions. Both studies were well conducted with good characterization of the participants and intention-to-treat analysis. However, the study from Rossignol et al. suffered from the absence of a placebo condition because it compared two different HBOT procedures. Recently, in 2012, another randomized trial [48] (which has a lower quality) has reported no significant difference between HBOT and a sham condition (with high pressure, but no supplemental oxygen). It is of note that both groups seemed to improve from baseline. A small open label trial reported improvement in several symptoms of ASD [46].

### 4.4. Chelation

Chelation treatment involves administration to an individual of various chemical substances for the purpose of binding and then withdrawing specific metals from the person's body [98]. Chelation in ASD has been investigated in a few studies collected in a review published in 2012 [129]. The review included 5 studies for a total of 82 participants. However, for the purpose of the present review, only the study from Adams et al. [49] and from D. A. Geier and M. R. Geier [50] could be included (the others were case report or case series) (Table 4). Adams et al. [49] conducted a double blind, randomized trial which did not demonstrate any significant evidence supporting the utility of chelation treatment in ASD. D. A. Geier and M. R. Geier [50] designed an open label trial in which children underwent chelation and antiandrogen therapy. The authors reported significant improvement, but the study design and the multicomponent intervention refrained to draw solid conclusions.

## 5. Nonbiologically Based CAM Treatments

The National Center for Complementary and Alternative Medicine (NCCAM) divides nonbiological CAM therapies in three groups: mind-body medicine (i.e., prayer, yoga, meditation, music, dance, and art in general), manipulative and body-based practices (i.e., massage, chiropractic care, and acupuncture), and energy medicine (i.e., Reiki or homeopathy) [112].

### 5.1. Music Therapies

Music therapy can be defined as “a systematic process of intervention wherein the therapist helps the client to promote health, sing musical experiences and the relationships that develop through them as dynamic forces of change” [130]. The role of music therapy as a treatment for several psychiatric conditions (i.e., depression, schizophrenia, substance dependence and abuse disorder, and dementia) has been studied for many years, because of its effectiveness in the domains of physical recovery, cognitive improvement, communication skills, and social and emotional rehabilitation [131]. Musical improvisation in autism could represent a sort of nonverbal shared language that could enable both verbal and nonverbal patients to reach communication [132]. In fact, it has been reported that the learning of language in infants is highly based on the musicality of sounds [133]. Additionally, children with ASD appeared to respond better to music than to spoken words [134]. As a hypothesis, because different brain regions processed music or words [133], the use of song could help people with ASD to understand emotion which they have difficulties in detecting in words.

The use of music therapy in the treatment of ASD patients has been tested in several studies (Table 5). In 2014 [135], the Cochrane Collaboration reviewed 10 randomized controlled trials (RCT) which have been published from 1995 to 2012 [51–60]. Considering all studies, total sample size was 93. The findings provided evidence that music therapy may help children with ASD to improve their skills in primary outcome areas like social interaction, verbal communication, initiating behavior, and social-emotional reciprocity. It may also help to enhance nonverbal communication skills within the therapy context. Furthermore, it may contribute to increasing social adaptation skills in children with ASD and to promoting the quality of parent-child relationships. However, several included studies suffered from a very small sample size and from difficulties in defining a standard in music therapy methodology in order to facilitate replicability. From this review, no other RCT have been published. We retrieved also two open label trials. Firstly, Boso and colleagues experimented music therapy in 8 adults with ASD. The study showed significant improvement in several standardized scales (CGI-S, CGI-I, and BPRS) [61]. Another recently published study included 10 patients. Iseri and colleagues did not find any change in hormonal levels but improvements in patients' behavior, social, and communication skills [62].

Other types of music-related therapies have been investigated in ASD, which involved music as an active part of the intervention. Kalas [63] tried to elicit responses to joint attention in 30 autistic children with ASD by making them listen to two different types of music (simple and complex). The cohort was divided into two groups of severity and the study demonstrated that while simple music was more effective in severe ASD patients' joint attention, complex music was more effective in children with mild or moderate autism. The main flaw of this study was the lack of a standardized outcome measure. Vibroacoustic music has been investigated in individuals with mental retardation and also in a small sample of patients affected by autism [64]. The randomized study was focused on self-injuring, aggressive and stereotypical behavior and demonstrated that vibroacoustic music may be useful in reducing these aspects. We have to underline that the study is not specific for ASD and that the experimenters did not account for possible confounders. Schwartzberg and Silverman [65] evaluated the social skills profile in children with autism in a three-week study. Children were divided into two groups: in one group the children were sung social stories, while in the other group the stories were simply read. Unfortunately, no significant differences between the two groups have been found. As in the previous studies, blindness is not reported. In addition, the drop-out rate is quite high and the authors only performed a per-protocol analysis.

### 5.2. Auditory Integration Training

Auditory Integration Training (AIT) involves a person listening to a selection of music which has been electronically modified. There are several kinds of AIT including the Berard Method, the Listening Program, the Samonas Sound Therapy, and the Tomatis Method [136]. AIT is based on the idea that some people, including some people with autism, are hypersensitive or hyposensitive to certain frequencies of sound. AIT is designed to improve the person's ability to process sounds by “re-educating” the brain [136].

The Cochrane Collaboration recently published a systematic review [136] that collects the main publications in this field of research (Table 6). Six relatively small studies [66–71] were included. All the experimental groups underwent two 30 min sessions of AIT for 10 consecutive days. The largest studies did not report a difference between treatment and control conditions and in one case there is no evidence for long-term benefits of AIT. These studies contained several flaws, like small sample sizes, wide range of participants' baseline characteristics (age and sex), unstandardized outcome measures, and blinding.

### 5.3. Sensory Integration Therapy

Individuals with ASD often display impairments in sensory information processing. As a result, situations involving contact with lights, sounds, smells, tastes, or textures could be overwhelming for patients [137]. Sensory integration therapy commonly uses play activities specifically studied to modulate how the brain responds to sight, touch, sound, and movement [138]. Even if common among families with an ASD child, its results have been controversial. We retrieved four trials [72–75] (Table 7). All studies yielded significant improvement in several autistic core symptoms (communication, social reciprocity, and motor activity). However, only two studies used a standardized form of sensory integration therapy to allow replicability. Additionally, only half of the trials used standardized outcome measures, while the others were based on direct observation of behaviors or ad hoc questionnaire. Another potential bias to consider is the lack of well-defined control group (some trials used fine motor activities such as tapping but with no additional details).

### 5.4. Drama Therapy

Drama represents a form of art, which could foster the development of social skills (i.e., pretention, communication, social reciprocity, and emotion recognition). Drama therapy may therefore represent a potential therapy for individuals with ASD [139]. In particular, one open label study (Table 8) investigated a specific form of theatrical therapy—SENSE (Social Emotional NeuroScience Endocrinology) theatre—specifically designed to ameliorate social functioning and stress in children with ASD [76]. The authors enrolled 8 autistic children and 8 matched normally developed subjects who would act as models for the ASD patients. The authors observed a mild improvement in Theory of Mind (ToM) skills and facial emotion recognition. Unfortunately, this study suffered from several flaws: the study design, the lack of a control group, and the small sample size.

### 5.5. Dance Therapy

Dance and movement therapy is an embodied treatment that uses mirroring of movements: each subject tries to mirror empathic movements of the therapist, focusing more on “attunement” than on simple imitation. This may represent the basis for more mature form of social reciprocity [140]. Mateos-Moreno and Atencia-Doña [77] (Table 8) evaluated the efficacy of a combination of dance/movement and music therapy in 8 children with ASD. The active group was compared to a control group: each participant additionally received specialized treatment for ASD (i.e., behavioral and pharmacological therapy). Both groups improved over time with a better profile for the active group. However, given the multicomponent intervention, it is difficult to define the single contribution of dance therapy. Additionally, this study presented several flaws: the open label design, the nonrandom selection of subjects, and the small sample size.

### 5.6. Acupuncture

Acupuncture (AP) is a form of Traditional Chinese Medicine [112], widely used also in Western countries. It consists in placing needles in the skin and near tissues in specific points, known as acupuncture points. The needle could convey also electricity (electro-AP) or laser or heat. Four randomized controlled trials were retrieved according to our inclusion criteria (Table 9). The first (2008) [78] used scalp acupuncture (which involved needles to be placed in specific locations such as ear, nose, hand, and foot). The authors randomly assigned 10 children to group A (language therapy without scalp acupuncture) and 10 children to group B (language therapy plus scalp acupuncture twice weekly for 9 months for a total of 50 sessions). Even if the authors observed an improvement in both groups in language, no statistical analysis comparing the two groups was performed. In 2009, seven-star needle stimulation (which used a dermatoneural hammer housing seven blunt needles forming the shape of a seven-point star) was tested in 16 children with ASD [79]. The investigators observed a significant improvement in EEG pattern and in parent-rated language and social communication. However, parents were not blinded to the intervention and each child was attending educational therapy as well. In 2010 two studies were designed and conducted. The first [80] enrolled 55 children with ASD who were assigned to either electro-AP plus conventional treatment or sham electro-AP plus conventional treatment for 4 weeks. There was an improvement in the Clinical Global Impression of change and in parent-rated score of social isolation, language, attention, and motor skills. Later on, the same author [81] randomized 50 children to tongue AP or sham tongue AP for 8 weeks. A significant improvement in the active group was reported. It is of note that the sham AP consisted in placing needles in nonacupuncture points and in the first trial by Wong and Chen sham AP also conveyed an electrical stimulation (so it was not properly sham).

### 5.7. Massage

Sensory hypo/hypersensitivity has become a symptom criterion for the diagnosis of ASD in the DSM-5 [141]. The use of touch in order to treat sensory impairment and reducing anxiety has been postulated in ASD [142]. A systematic review has recently investigated the effect of massage in ASD [143]. We retrieved four single blind, randomized trials which examined different types of massage (from simple touch to Thai massage) (Table 10). One study did not use standardized measures of outcome. Among the others, the brief report from Escalona et al. [82] involved 20 children who were assigned to touch therapy (15 min daily) provided by parents at bedtime or a control group (parents read bedtime stories to their child) for one month; the touch group showed improved social relatedness and a reduction in stereotyped behaviors. Two studies from Silva et al. [83, 84] evaluated a massage technique (the Qigong, consisting in massage manipulation from head to foot along acupuncture channels, lasting for 15 minutes). The first report [83] involved 13 children with ASD, randomly assigned to Qigong daily for five months plus a special education program or to the special education program alone. Children in the active group showed significant improvement in sensory impairment and Vineland Daily Living and Socialization subscores compared to controls. The second randomized trial [84] involved a larger sample with the same study protocol. Significant increase in socialization and communication and a reduction of sensory impairment were observed. More recently, another type of massage therapy (Thai massage) [85] was randomly administered to 60 children with ASD. The active group received Thai massage plus sensory integration therapy while the control group underwent sensory integration therapy alone. Thai massage was provided twice weekly for 8 weeks. At the end of the trial, the Thai massage group showed reduced anxiety and conduct problems measured through standardized parent-rated scales.

### 5.8. Yoga

Yoga is a movement therapy which could potentially ameliorate behavioral problems and anxiety (Table 11). It is of note that yoga appears to increase GABA brain levels, even after one session [144]. As GABA is considered to play a key role in autism pathogenesis, yoga may in theory represent a potential treatment candidate. In 2011, Rosenblatt et al. [86] conducted a pilot study in which they investigated combined yoga, dance, and music therapy in 24 children with ASD. The program consisted in 8 sessions of this technique: study findings showed no significant difference in the primary outcome measure—the Aberrant Behavioral Checklist (ABC) Irritability subscale. In 2012, a school based “get ready to learn Yoga” program was tested for efficacy in 48 children with autism [87]. The intervention was a manualized yoga technique performed by the teachers daily for 16 weeks, while the control group attended standard school morning activities. The authors found a significant reduction in teacher-rated ABC scores in the intervention group. Another movement therapy consisted in a mind-body exercise: 46 children with ASD were randomly assigned to either active treatment (Nei Yang Gong, a Chinese technique which “emphasizes the maintenance of a natural and relaxed attitude to achieve smooth circulation of Qi and blood” and involved “simple body movement” which has to be performed in a “relaxed and natural manner”) or control treatment (Progressive Muscle Relaxation) [88]. Overall, the Nei Yang Gong session lasted for 5 minutes, while the control treatment session lasted for 20 minutes and were done twice per week for four weeks. Each participant was advised to practice also at home. Study findings reported increased self-control, reduced parent-rated autistic symptoms, and increased control of disruptive behaviors. In the Nei Yang Gong group, subjects displayed greater EEG activity in the anterior cingulate cortex.

### 5.9. Pet Therapy

The use of animals in ASD relies on the hypothesis that animal movements and behaviors are more predictable and repetitive and could help children with ASD to interpret social cues even in more subtle contexts (Table 12) [145]. The first studies conducted on pet therapy used dogs but were flawed by the absence of any type of standardized clinical measure or a lack of diagnostic characterization of subjects. Later on, more methodologically sounded trials were designed: in 2011, equine-assisted pet therapy was evaluated with an open label, prospective trial [89]. Twenty-four children with ASD entered a 3-to-6-month waiting list and subsequently switched to the horse riding treatment for 6 months. Only twenty children completed the trial. The results showed a significant reduction of CARS scores during the riding period compared to the waiting list. In 2014, Guinea pigs were used with 64 children with ASD [90]. The study has a nested design (classrooms in schools) and the animal therapy group was compared to the waiting list. The authors reported significant improvement in social functioning compared to the control situation.

### 5.10. Chiropractic Care

Chiropractic care is a popular and widely used CAM in ASD [146]. Its use in patients with ASD has been considered in a systematic review written by Alcantara et al. [147]. Beside three case reports, the review contained one cohort study [91] and one randomized comparison trial [92] (Table 13). In the first study, a cohort of 26 autistic children received chiropractic care for 9 months. Behavioral symptoms, based on parent and teacher assessments, improved. Additional improvements included decreased medication use. However, this study presents several biases, first of all the lack of a control group and the small sample size. The experiment conducted by Khorshid and colleagues compared two different types of chiropractic care: the Atlas Orthogonal Upper Cervical Spinal Manipulative Therapy (which is a form of chiropractic manipulation involving the instrumental percussion of the atlas to correct possible misalignments) versus full-spine Spinal Manipulative Therapy (which is characterized by high velocity and low amplitude thrusts) in children with autism. The major improvement was found in the Atlas Orthogonal Group. However, there is an evident flaw in the study, that is, the lack of a control group.

## 6. Discussion

In recent years, CAM therapies have gained attention by the scientific community: several studies have been conducted in order to investigate the efficacy and safety of CAMs in ASD. We reviewed trials on different CAMs, but the findings are still inconclusive. In particular, there is a lack of proof regarding the efficacy of CAM in autism. It is of interest that it is striking the contrast between the wide use of CAM by families and the paucity of scientific results for alternative treatments. One possible reason for this discrepancy is that CAM therapies are usually considered as “natural,” with an optimal safety profile and less side effects than conventional medications [8]. This is partly true, as nonbiological CAMs are virtually free from adverse events; unfortunately, however, several alternative treatments are prone to safety issues, such as chelation or high doses of vitamins. Additionally, even if no serious adverse events were recorded in the revised trials, some treatments as elimination diets could be associated with potentially harmful, long-term side effects, such as nutritional deficits in children with higher food selectivity. In fact, CAM appears to be safe in the short period, but no data are available for longer treatment.

Considering the reviewed treatments, among biologically based CAMs only gluten/casein-free diet, omega 3, vitamin supplementation (vitB6, vitB12, and tetrahydrobiopterin), and Hyperbaric Oxygen Therapy have been more extensively studied: all the other biological therapies were tested in single trials and therefore provided no sufficient data in order to determine their usefulness in clinical practice. Elimination diet does not appear effective in treating ASD core symptoms: the fact that individual patients may benefit from special dietary interventions could be hypothesized as the result of subclinical intolerance to specific food allergen [111]. Omega 3 supplementation provided no evidence for recommendation in ASD: the only positive results come from a single open label trial [26]. Trials evaluating vitamin supplementation yielded inconsistent results: as all studies presented several caveats, more data should be obtained before definitive judgment. Additionally, for instance, vitB12 should be administered through injection, thus potentially reducing compliance [32]. Hyperbaric Oxygen Therapy has been only recently scientifically tested: study findings are promising but not completely consistent. Future studies with larger sample size, well-designed randomization, blindness, and definition of a placebo condition will be needed.

Among nonbiologically based CAMs, the treatments more extensively investigated are music therapy, Auditory Integration Training, Sensory Integration Therapy, acupuncture, and massage. No sufficient data are available for several interventions such as dance therapy, drama therapy, or pet therapy. It is of note that music therapy is not always regarded as a CAM treatment but considered as a part of behavioral intervention [10]: this could explain the relatively high number of studies on music compared to other CAMs. Promising evidence supports the use of music in children with ASD, which seems to impact several symptom domains such as communication, social reciprocity, and emotion. Additionally, music therapy and all nonbiological CAM treatments appear extremely safe with no side effects. Results from Auditory Integration Training studies are conflicting: more trials should be designed to better elucidate the findings; in particular, more attention should be given to blindness of investigators and assessors and to the choice of widely used standardized outcome measures. Evidences from Sensory Integration Therapy, acupuncture, and massage cautiously support the use of these treatments in clinical care: however, there are several flaws that should prevent overinterpretation of the findings (small sample sizes, unclear blinding of the assessors, lack of a defined placebo condition, and multicomponent intervention).

Overall, there is sparse evidence on the usefulness of CAM treatments in ASD. A potential explanation for these unclear results is that well-designed studies have only recently been developed and usually have limited sample size. Moreover, the heterogeneous nature of ASD and the presence of possible comorbidities could have impaired several trials which lacked a correct stratification of participants. Interestingly, almost all the reviewed trials were focused on children with ASD: as prevalence rates of ASD are increasing constantly, more adults each year are confronting the challenges of autism. Thus it will be interesting to test CAM therapies in an adult population.

We advise practitioners to encourage patients and their families to discuss the efficacy and safety of all CAMs. Patients must be informed of possible interactions between CAMs and currently prescribed drugs. Clinicians should allow families or patients to try CAMs with limited clinical evidence if they are safe and cheap and if they do not prevent patients from obtaining evidence-based treatments (i.e., behavioral therapies). It is of note that several CAMs could be easily used together with standard clinical care: in particular, nonbiologically based CAMs (i.e., music therapy, pet therapy) could be added to conventional treatment, not as a replacement but as an augmentation or implementation of standard therapy. For instance, massage or music could reduce anxiety and enhance positive response to behavioral and educational treatments. Practitioners should advise patients to try one CAM at a time and should constantly monitor clinical changes and adverse events.

In conclusion, there are still few data on the potential efficacy of CAM in autism, and no evidence-based recommendation could be done so far for the use of such therapies. To shed more light on CAM efficacy in autism, large randomized controlled trials with a better characterization of patients are needed.

## Figures and Tables

**Table 1:** Dietary intervention in ASD.

Author	Year	Type and duration of study	Sample size	Type of intervention	Comparators	Outcome measure	Findings	Comments
Knivsberg et al. [13]	2002	Randomized, placebo controlled, single blind, parallel group Duration: 12 months	*n = *20 (gender not reported) Age: 59–127 months	Gluten- and casein-free diet (GFCF) *n = *10	Normal diet *n* = 10	DIPAB (a Danish assessment of autistic trait), Leiter International Performance Scale, ITPA, Reynells språktest, Movement Assessment Battery for Children	Significant improvement in all domains for the diet group compared to the control group	Parent not blinded to diet Small sample size Not standardized assessment of autistic traits No accounting for potential confounders (medication, other therapies) Few baseline characteristics for patients No assessment of dietary fidelity

Elder et al. [14]	2006	Randomized, double blind, repeated measures, crossover Duration: 12 weeks	*n = *15 (M 12; F 3) Age: 2–16 years	GFCF	Matched diet but with gluten and casein	CARS Urinary Peptide Levels, ECO Language Sampling Summary, behavioral observation by at home videos (at week 6 and week 12)	No significant differences between the two groups	Small sample size High heterogeneity in patients Short study duration Dietary fidelity not always adequate Missing data for some variables No accounting for potential confounders (medication, other therapies)

Whiteley et al. [15]	2010	Randomized, double blind, placebo controlled, partly crossover (at 12 months, not responders in the control group switch to diet) Duration: 24 months	*n = *72 (gender not reported) Age: 4–11 years	GFCF (*n = *38) Drop-out at 12 months *n = *11, one patient removed additionally for protocol deviation Analysis at 12 months carried out on 26 children (M 21; F 5) Analysis at 24 months carried out on 18 children	Normal diet (*n* = 34) Drop-out at 12 months *n* = 4, one patient removed additionally for protocol deviation Analysis at 12 months carried on 29 children (M 28; F 1) Analysis at 24 months carried out on 17 children	ADOS-G, VABS, ADHD-IV, and GARS (at baseline and 8–12–20–24 months)	Significant improvement in the diet group at 12 and 24 months in ADOS-communication and repetitive domains, GARS social domains	Parent not blinded to diet Sample size seems adequate but not power analysis provided Only per-protocol statistical analysis High attrition rate Study design and crossover not completely clear No accounting for potential confounders (medication, other therapies) No assessment of dietary fidelity

Johnson et al. [16]	2011	Randomized, parallel groups Duration: 3 months	*n = *22 (M 18; F 4) Age: 3–5 years Diagnosis of ASD only in 20 patients, PDD-NOS in 2 patients	GFCF *n* = 8	Low sugar healthy diet *n* = 14	Mullen Scales of Early Learning, CBC, direct observation of behavior (at baseline and after 3 months)	No significant clinical difference between the two groups (improvement in CBC aggression and CBC ADHD in GFCF group)	Blinding not reported (parent not blinded) Small sample size Low dietary adherence in GFCF group No accounting for potential confounders (medication, other therapies)

Evangeliou et al. [17]	2003	Prospective, open label Duration: 6 months	*n* = 30 (M 16; F 14) Drop-out *n* = 7 Age: 4–10 years	Ketogenic diet according to John Radcliffe (30% medium-chain triglyceride oil, 30% fresh cream, 11% saturated fat, 19% carbohydrates, and 10% proteins) was administered for 6 months, with intervals of 4 weeks interrupted by two diet-free weeks	None	CARS	Improvement	Low dietary tolerance and subsequent high attrition rate Small sample size Open label trial Assessors not blinded Statistical analysis not optimal All patients were taking haloperidol

Karkelis et al. [18]	2010	Randomized, placebo controlled, parallel group Duration: 4 months	*n* = 45 (gender not reported) Age: 2–8 years IgE positive for milk allergy *n = *17	Elemental formula diet (containing free amino acids) with no milk product *n* = 22 (positive for milk allergy *n* = 9)	Normal diet *n* = 23 (positive for milk allergy *n* = 8)	Hyperactivity	Significant improvement in hyperactivity in the elemental diet for patients with milk allergy and ASD without food allergy	Blinding not reported Baseline data not complete Preliminary report Not standardized outcome measure Sample size seems adequate but no power analysis

Chan et al. [19]	2012	Randomized, double blind, parallel group Duration: 1 month	*n* = 24 (M 20; F 4) Age: 7–17 years	Chan diet *n* = 12	Normal diet *n* = 12	ATEC, Five-Point Test, Tower of California, go/no go task, D2 Test of Concentration, CCTT	Significant improvement in ATEC in the experimental group	Blinding not reported (parents appear not blinded, which could alter ATEC) Small sample size Short study duration Statistical analysis not optimal

Al-Ayadhi and Elamin [20]	2013	Randomized, double blind, placebo controlled, parallel group Duration: 2 weeks	*n* = 60 (gender not reported) Age: 2–12 years	Camel milk raw (*n* = 24) or boiled (*n* = 25)	Cow milk as placebo *n* = 11	CARS	Significant improvement in CARS after introduction of camel milk	Short study duration Statistical analysis not optimal Few baseline characteristics of patients Number of patients not balanced between groups

Bashir and Al-Ayadhi [21]	2014	Randomized, double blind, placebo controlled, parallel group Duration: 2 weeks	*n* = 45 (M 40; F 5) Age: 2–12 years	Camel milk raw (n = 15) or boiled (*n* = 15) Additionally 6 patients dropped out (4 in the boiled group and 2 in the raw)	Cow milk as placebo *n = *15 Additionally 3 patients dropped out	CARS	Significant improvement in CARS in the raw camel milk group	Short study duration Only per-protocol analysis High drop-out rate

ADHD-IV, Attention-Deficit Hyperactivity Disorder-IV rating scale; ADOS, Autism Diagnostic Observation Schedule; ATEC, Autism Treatment Evaluation Checklist; ASD, autism spectrum disorder; CARS, Childhood Autism Rating Scale; CBC, Child Behavior Checklist; CCTT, Children's Color Trails Test; ECO, Ecological Communication Orientation; GARS, Gilliam Autism Rating Scale; GFCF, gluten- and casein-free diet; ITPA, Illinois Test of Psycholinguistic Abilities; PDD-NOS, Pervasive Developmental Disorder Not Otherwise Specified; VABS, Vineland Adaptive Behavior Scale.

**Table 2:** Nutraceuticals in ASD.

Author	Year	Type and duration of study	Sample size	Type of intervention	Comparators	Dose	Outcome measure	Findings	Comments
Omega 3									

Amminger et al. [22]	2007	Randomized, double blind, placebo controlled, parallel group Duration: 6 weeks	*n* = 13 (M 13) Age: 5–17 years	Omega 3 *n* = 7	Placebo *n* = 6 One drop-out from this group	0.84 g/day EPA and 0.7 g/day DHA	ABC	No significant difference between the two groups	No details about blinding Small sample size Power analysis not reported

Bent et al. [23]	2011	Randomized, double blind, placebo controlled, parallel group Duration: 12 weeks	*n* = 27 (M 24; F 3) Age: 3–8 years	Omega 3 *n* = 14 Drop-out *n* = 1	Placebo *n* = 13 Drop-out *n* = 1	0.7 g/day EPA and 0.46 g/day DHA	ABC, PPVT-III, EVT, SRS, BASC, CGI-Improvement	No statistically significant difference	Only per-protocol analysis Small sample size Short study duration

Bent et al. [24]	2014	Randomized, double blind, placebo controlled, parallel group Duration: 6 weeks	*n* = 57 (M 50; F 7) Age: 5–8 years	Omega 3 *n* = 29	Placebo *n* = 28	1.3 g/daily of omega 3 (1.1 g/day of EPA plus DHA)	ABC, CGI, SRS	No statistically significant difference	Small sample size Short study duration

Meguid et al. [25]	2008	Open label Duration: 3 months	*n* = 30 (M 18; F 12) Age: 3–11 years	Omega 3	None	240 mg/day DHA; 52 mg/day EPA; 68 mg/day Omega-6 fatty acids	CARS	Improvement in 20 children	Statistical analysis incorrect (conducted only on children which showed a reduction in symptoms) Open label design Small sample size Multicomponent intervention (also omega 6)

Politi et al. [26]	2008	Open label Duration: 6 weeks	*n* = 19 (M 50; F 7) Age: 18–40 years	Omega 3	None	0.93 g/day EPA plus DHA, 5 mg/day vitamin E	Rossago behavioral checklist	No significant change from baseline	Small sample size Short study duration Open label design No standardized outcome measure

Voigt et al. [27]	2014	Randomized, double blind, placebo controlled, parallel group Duration: 6 months	*n* = 48 (M 40; F 8) Age: 3–10 years	Omega 3 *n* = 24	Placebo *n* = 24	200 mg/day DHA	CGI-Improvement scale, CDI, ABC, BASC	No significant difference between active group and placebo	Small sample size Low dosage

Vitamins									

Findling et al. [28]	1997	Randomized, double blind placebo controlled, crossover Duration: 8 weeks	*n* = 12 (M 11; F 1) Drop-out *n* = 2 Age: 3–12.9 years	Vitamin B6-magnesium	Placebo	B6: 30 mg/kg/day (max = 1 g/day); Mg: 10 mg/kg/day (max = 350 mg/day)	CARS, CGI, CPRS, OCS	No difference between the two groups	Small sample size Short study duration Multicomponent intervention Per protocol analysis

Kuriyama et al. [29]	2002	Randomized double blinded placebo controlled parallel group Duration: 4 weeks	*n* = 15 Entered the study only *n* = 8 (M 4; F 4) Drop-out *n* = 7 Age: 3–12.9 years	Vitamin B6 *n* = 4	Placebo *n* = 4	B6: 200 mg/day	IQ (WISC-III) and SQ (SM)	No difference between active group and placebo	Small sample size High attrition rate Unclear characterization of patients “children with PDDs who exhibit clinical features similar to those of pyridoxine dependent epilepsy but do not have a history of seizures” Clinical characterization unclear (only PDD)

Tolbert et al. [30]	1993	Randomized double blind placebo controlled, asymmetric crossover with 10 week treatment blocks (B6/Mg 20 w-placebo 10 w; or B6/Mg 10 w-placebo 10 w-B6/Mg 10 w) Duration: 30 weeks	*n* = 15 (M 10; F 5) Age: 6–18 years	Vitamin B6-magnesium	Placebo	B6: 200 mg/70 kg; Mg: 100 mg/70 kg	RLRS	No difference	No information on placebo Presence of a control group, but not randomized Small sample size Low dosage of B6/Mg Asymmetric study design

Bertoglio et al. [31]	2010	Randomized, double blind, placebo controlled, crossover Duration: 12 weeks	*n* = 30 (M 28; F 2) Age: 3–8 years	Methyl B12	Placebo	64.5 mcg/kg every three days	CGI-I, PIA-CV, CARS, PPVT-III, ABC, CBC, Stanford Binet Fifth Edition Routing Subsets, MCDI	No significant difference between active treatment and placebo	Small sample size Short study duration Improvement only in post hoc analysis

Frye et al. [32]	2013	Open label trial Duration: 3 months	*n* = 48 (gender not explicitly reported) Drop-out *n* = 11 Age: 4–6 years	Methyl B12 vitamin plus folinic acid	None	75 *μ*g/Kg methylcobalamin every three Folinic acid (400 *μ*g) twice daily	Vineland	Improvement in all Vineland subscales	Only patients with abnormal redox metabolism were included High attrition rate Statistical analysis only performed on completers Small sample size

Dolske et al. [33]	1993	Randomized double blinded placebo controlled, crossover with asymmetric design with 10 w block (randomly assigned to vitC-vitC-placebo or vitC-placebo-vitC) Duration: 30 weeks	*n* = 18 Age: 3–8 years	Vitamin C	Placebo	8 g/70 kg/day	RLRS	Significant improvement in vitC treated	Small sample size Short study duration

Adams et al. [34]	2011	Randomized double blinded placebo controlled trial Duration: 3 months	*n* = 141 (M 125; F 16) Age: 5–16 years	Multivitaminic supplement *n* = 72 Drop-out *n* = 19	Placebo *n* = 69 Drop-out *n* = 18	Each supplement was titrated to be over the recommended daily allowance but under the tolerable upper limit	PDD-BI, ATEC, SAS, parent-rated behaviors	Improvement of parent-rated irritability in the active group	No power calculation High attrition rate Only per-protocol analysis

Danfors et al. [35]	2005	Randomized, double blind, placebo controlled, crossover Duration: 6 months	*n* = 12 (M 12) Age: 4–7 years	Tetrahydrobiopterin (BH4)	Placebo	3 mg/kg/day	CARS (baseline, 3 and 6 month)	Small changes in CARS total score	Small sample size Only post-hoc analysis reveals significant changes in CARS subdomain

Frye et al. [36]	2013	Open label Duration: 16 weeks	*n* = 10 (M 9; F 1) Age: 2–6 years Children must have low cerebrospinal fluid level of BH4	Tetrahydrobiopterin (BH4) Drop-out *n* = 2	None	20 mg/kg/day	PLS, SRS, CARS, ASQ, Vineland	Significant improvement in PLS, CARS, ASQ, and Vineland	Open label Small sample size High attrition rate (20%) High tetrahydrobiopterin dose (usually 1–6 mg/kg/day)

Klaiman et al. [37]	2013	Randomized, double blind, placebo controlled, parallel group Duration: 16 weeks	*n* = 46 Age: 3–7 years	Tetrahydrobiopterin (BH4)	Placebo	20 mg/kg/day	CGI, PLS, ABC, SRS, Vineland	No significant difference between the two groups	Improvement only in post hoc analysis on secondary measures High tetrahydrobiopterin dose (usually 1–6 mg/kg/day)

L-carnosine									

Chez et al. [38]	2002	Randomized double blinded placebo controlled Duration: 8 weeks	*n* = 31 (M 21; F 10) Age: 3–12 years	L-Carnosine *n* = 17	Placebo *n* = 14	800 mg/day	CARS, GARS, CGI Expressive and Receptive One-Word Picture Vocabulary tests,	Significant improvement in the active group in GARS and in the Receptive One-Word Picture Vocabulary test	Small sample size No power calculation Half of the subjects were on stable dosage of valproic acid

Flavonoids									

Taliou et al. [39]	2013	Open label Duration: 26 weeks	*n* = 50 (M 42; F 8) Drop-out *n* = 10 Age: 4–10 years	Flavonoid 1 capsule/10 Kg/day	None	Luteolin (100 mg/capsule), quercetin (70 mg/capsule), and the rutin (30 mg/capsule)	Vineland, ABC, ATEC, CGI-improvement	Improvement in Vineland and ABC scores	Open label design Small sample size High attrition rate

Probiotics									

Kałuzna- Czaplińska and Błaszczyk [40]	2012	Open label Duration: 2 months	*n* = 22 (M 20; F 2) Age: 4–10 years	Probiotic 1 capsule twice daily	None	*Lactobacillus acidophilus* (strain Rosell-11, containing 5 × 10^9^ CFU/g)	Observer rated autism core symptoms	Improvement	Open label design All children have gastrointestinal problems Other CAM treatments were used concomitantly Small sample size No standardized outcome measure

Enzyme supplementation									

Munasinghe et al. [41]	2010	Randomized double blinded placebo controlled crossover Duration: 6 months	*n* = 43 (M 36; F 7) Drop-out *n* = 16 Age: 3–8 years	Digestive enzyme supplement	Placebo	Peptizyde (Peptidase, Protease 4.5 and Papain) two capsules with each meal	GBRS, ARS of gastrointestinal symptoms, and the Rescorla LDS	No difference	High drop-out rate Not clear if intention-to-treat or per-protocol analysis Not all outcome measures were standardized

Herbal remedies									

Hasanzadeh et al. [42]	2012	Randomized double blinded placebo controlled parallel group Duration: 10 weeks	*n* = 47 (M 39; F 8) Age: 4–12 years All children were on risperidone 2-3 mg/day according to weight	*Ginkgo biloba n* = 23 (M 19; F 4)	Placebo *n* = 24 (M 20; F 4)	80 mg/day if weight <30 kg; otherwise 120 mg/day	ABC	No difference	Small sample size Short study duration Only one outcome measure

Miyaoka et al. [43]	2012	Open label Duration: 12 weeks	*n* = 40 (M 22; F 18) Age: 8–40 years	Yokusan	None	5.0–7.5 g/day	CGI-Severity, ABC	Improvement in CGI and the irritability subscale of ABC	Open label design Small sample size

Chan et al. [44]	2014	Open label Duration: 6 months	*n* = 30 (M 26; F 4) Age: 7–17 years	Bornel and Borax nasal drops *n* = 15	None *n* = 15	10 mL/day	BRIEF, Tower of California, CCTT, CPRS, event-related EEG assessment	Improvement	Not placebo controlled Open label design Small sample size Statistical analysis not optimal

ABC, Aberrant Behavior Checklist; ARS, Additional Rating scale; ATEC, Autism Treatment Evaluation Checklist; ASD, autism spectrum disorder; ASQ, Autism Symptoms Questionnaire; BASC, Behavioral Assessment System for Children; BRIEF, Behavior Rating Inventory of Executive Function; CARS, Childhood Autism Rating Scale; CCTT, Children's Color Trails Test; CDI, Child Development Inventory; CGI-I, Clinical Global Impression Scale of Improvement; CPRS, Children's Psychiatric Rating Scale; DHA, docosahexaenoic acid; EPA, eicosapentaenoic acid; EVT, Expressive Vocabulary Test; GARS, Gilliam Autism Rating Scale; GBRS, Global Behaviour Rating Scale; IQ, Intelligence Quotient; LDS, Language Development Survey; OCS, Obsessive Compulsive Scale; PDD-BI, Pervasive Development Disorder Behavior Inventory; PLS, Preschool Language Scale; PPVT-III, Peabody Picture Vocabulary Test-Third Edition; RLRS, Ritvo-Freeman Real Life Rating Scale for Autism; SAS, Severity of Autism Scale SM; Social Maturity; SQ, Social Quotient; SRS, Social Responsiveness Scale; VABS, Vineland Adaptive Behavior Scale; WISC-III, Wechsler Intelligence Scales for Children-III.

**Table 3:** Hyperbaric Oxygen Therapy in ASD.

Author	Year	Type and duration of study	Sample size	Type of intervention	Comparators	Dose	Outcome measure	Findings	Comments
Granpeesheh et al. [45]	2010	Randomized, double blind, placebo controlled, parallel group Duration: maximum 15 weeks	*n* = 46 (gender not reported) Age: 2–14 years Drop-out *n* = 12 Patients controlled for medication and educational therapy	HBOT (1.3 atm and supplemental oxygen approximately 24–28% FiO_2_) Completers (*n* = 18)	Placebo Completers (*n* = 16)	80 1-h sessions	ABC, ADOS, CGI, Vineland, SRS, BRIEF, PSI, PPVT-III, VMI	No significant difference	High attrition rate

Rossignol et al. [46]	2007	Open label Duration: 4 weeks	*n* = 18 (M 14; F 4) Age: 3–16 years	HBOT (1.5 atm and supplemental oxygen (100% FiO_2_), *n* = 6 HBOT (1.3 atm and supplemental oxygen (24% FiO_2_), *n* = 12 Drop-out *n* = 4	None	40 1-h sessions	ATEC, ABC-community, SRS	Significant improvement in both groups in ATEC and SRS	Open label Small sample size

Rossignol et al. [47]	2009	Randomized, double blind, parallel group Duration: 4 weeks	*n* = 62 (M 52; F 10) Age: 2–7 years	HBOT (1.3 atm and supplemental oxygen (approximately 24% FiO_2_), “active group,” *n* = 33 Drop-out *n* = 4	HBOT (1.03 atm and supplemental oxygen (approximately 21% FiO_2_), “control group,” *n* = 33 Drop-out *n* = 3	40 1-h sessions	ATEC, CGI, ABC	Significant improvement in the active group in CGI receptive language, social interaction, and eye contact. Improvement in ATEC sensory/cognitive awareness	No placebo

Sampanthavivat et al. [48]	2012	Randomized, double blind, parallel group Duration: unclear	*n* = 60 Age: 3–9 years	HBOT (1.5 atm and supplemental oxygen 24% FiO_2_), *n* = 33 Drop-out *n* = 4	Sham air (1.15 atm), *n* = 33 Drop-out *n* = 3	20 1-h sessions	ATEC, CGI-change, and CGI-severity	No differences between the two groups	No placebo

ABC, Aberrant Behavior Checklist; ADOS, Autism Diagnostic Observation Schedule; ATEC, Autism Treatment Evaluation Checklist; ASD, autism spectrum disorder; BRIEF, Behavior Rating Inventory of Executive Functioning; CGI, Clinical Global Impression; PPVT-III, Peabody Picture Vocabulary Test-Third Edition; PSI, Parent Stress Index; SRS, Social Responsiveness Scale; VABS, Vineland Adaptive Behavior Scale; VMI-5, Beery-Buktenica Developmental Test of Visual-Motor Integration—5th edition.

**Table 4:** Chelation in ASD.

Author	Year	Type and duration of study	Sample size	Type of intervention	Comparators	Dose	Outcome measure	Findings	Comments
Adams et al. [49]	2009	Randomized, double blind, placebo controlled, parallel group Duration: 3 days	*n* = 77 (M 69; F 8) Age: 3–8 years Each subject was subjected to one round of DMSA to eliminate low metal excretor	Dimercaptosuccinic acid (DMSA) therapy, Completers *n* = 26	Placebo, Completers *n* = 15	Sic round of DMSA in three days	ATEC, PDD-BI, SAS, ADOS, PGI	No significant differences between active treatment and control	Very high attrition rate No power calculation Not real placebo (each subjects in the placebo group received a round of DMSA) Multicomponent intervention (patients received before enrollment glutathione)

D. A. Geier and M. R. Geier [50]	2006	Open label Duration: minimum 2–maximum 7 months	*n* = 11 (M 10; F 1) Age: 6–14 years	(1) Meso-2,3-dimercaptosuccinic acid (2) Leuprolide acetate	None	(1) 10 mg/kg twice daily (2) 15 mg every 28 days	ATEC	Significant improvement	Open label Small sample size Multicomponent intervention Potential conflict of interest

ADOS, Autism Diagnostic Observation Schedule; ATEC, Autism Treatment Evaluation Checklist; ASD, autism spectrum disorder; PDD-BI, Pervasive Developmental Disorder-Behavior Inventory; PGI, Parent Global Impressions; SAS, Severity of Autism Scale.

**Table 5:** Music therapies in ASD.

Author	Year	Type and duration of study	Sample size	Type of intervention	Comparators	Dose	Outcome measure	Findings	Comments
Arezina [51]	2011	Randomized, crossover Duration: 5 weeks	*n* = 6 (M 5; F 1) Age: 36–64 months	Interactive MT (musical instrument play, songs, music, books)	(1) Nonmusic interactive play (nonmusic toys and books) (2) Independent play	18 sessions of 10 minutes each	Behavior observation of videotaped sessions	Significant more interactions during interactive music therapy than the two comparator groups. Significant more requesting during interactive than independent play, but no effect of music	Blinding not reported No details about diagnostic process No baseline assessment of functioning Small sample size No standardized outcome measures No accounting for potential confounders (medication, other therapies)

Brownell [52]	2002	Randomized, crossover Duration: 4 weeks	*n* = 4 (M 4; F 0) Age: 6–9 years	Structured receptive MT (songs with social stories)	(1) Structured receptive “story therapy” (reading of social stories) (2) No intervention, (5 days)	5 individual daily sessions	Repetitive behaviors outside therapy sessions (in classroom)	No difference	Blinding not reported No details about diagnostic process Small sample size No standardized outcome measures No accounting for potential confounders (medication, other therapies)

Buday [53]	1995	Randomized, single blind, crossover Duration: 2 weeks	*n* = 10 (M 8; F 2) Age: 4–9 years	Structured receptive MT (songs used to teach signs)	“Rhythm therapy” (rhythmic speech used to teach signs)	5 individual sessions	Imitating behavior in sessions (sign and speech imitation)	Significant improvement of imitation in the music versus rhythmic conditions	Small sample size No details about diagnostic process No standardized outcome measures No accounting for potential confounders (medication, other therapies)

Farmer [54]	2003	Randomized, parallel group Duration: 5 days	*n* = 10 (M 9; F 1) Age: 2–5 years	Music therapy sessions (combined active and receptive: guitar playing, songs), *n* = 5	Placebo (no music) sessions, *n* = 5	5 individual sessions of 20 minutes	Responses within sessions: (a) verbal responses, (b) gestural responses	Significant increase in verbal responses in the music group versus placebo. No significant difference in gestural responses	Blinding not reported No details about diagnostic process Small sample size No standardized outcome measures No accounting for potential confounders (medication, other therapies) Number of subjects per session varies

Gattino et al. [55]	2011	Randomized, single blind, parallel group Duration: 7 months	*n* = 24 (M 24; F 0) Age: 7–12 years	Relational music therapy (improvisation not using a structured protocol) plus standard care, *n* = 12	Standard treatment (clinical routine activities), *n* = 12	20 thirty-minute sessions, scheduled weekly	CARS, Brazilian version	No statistical difference between the two groups. Subgroup analysis on nonverbal communication showed improvement in the music group	Single blind Small sample size No accounting for potential confounders (medication, other therapies)

Kim et al. [56]	2008	Randomized, single blind, crossover Duration: 8 months	*n* = 15 (M 13; F 2); drop-out *n* = 5 (M 3; F 2) Age: 39–71 months Diagnosis of ASD by two child psychiatrists	Improvisational music therapy	Play sessions with toys	12 thirty-minute sessions, scheduled weekly	PDD-BI, ESCS, eye contact frequency and duration, initiation of engagement frequency, emotional synchronicity frequency and duration, musical synchronicity frequency and duration, number of compliant-no compliant and absent responses, joy frequency and duration	Significant improvement only in ESCS score after music therapy compared to play (medium effect size). Eye contact was longer in music therapy than in play	Single blind (additionally, assessors were not blinded to all outcome measures, in particular to ESCS) Small sample size No accounting for potential confounders (medication, other therapies) High drop-out rate Statistical analysis performed only in completers

Lim [57]	2010	Randomized, single blind, parallel group Duration: 5 days	*n* = 50 (M 44; F 6) Age: 3–5 years	Music training (“Developmental Speech and Language Training through Music”; videotaped songs with target words), *n* = 18	(1) Speech training (videotaped spoken stories with target words), *n* = 18 (2) No training, *n* = 14	6 individual sessions within 3 days	Behavior observation (verbal response) of videotaped posttest sessions	No differences between music and speech therapy (improvement in both groups versus no treatment). Higher improvement in low functioning children	Single blind No standardized outcome measures No details about the diagnostic process No accounting for potential confounders (medication, other therapies)

Lim and Draper [58]	2011	Randomized, single blind, crossover Duration: 2 weeks	*n* = 22 (M 17; F 5) Age: 3–5 years	Applied Behavior Analysis Verbal Behavior plus Music Training (sung instructions, songs with target words)	(1) Applied Behavior Analysis Verbal Behavior (2) No training	6 individual sessions within 2 weeks	Behavior observation (verbal production) of videotaped posttest sessions	No statistically significant difference between the two treatment groups	Single blind Small sample size No standardized outcome measures No details about the diagnostic process No accounting for potential confounders (medication, other therapies)

Thomas and Hunter [59]	2003	Randomized, crossover Duration: 12 weeks	*n* = 6 (M 5; F 1) Age: 2-3 years	Music therapy (songs, instruments, vocal sounds, and movement to interact with the child, musical or verbal response to the child's behavior)	Playtime (interact with the child using toys and verbal response to the child's behavior)	Twelve 15-minute sessions	Behavior observation (on-task and requesting) of videotaped sessions, assessed as percentage of session time	Significant improvement in social adaptation and initiating behaviors in the music compared to play	Blinding not reported Small sample size No standardized outcome measures No accounting for potential confounders (medication, other therapies)

Thompson [60]	2012	Randomized, parallel group Duration: 12 weeks	*n* = 23 (M 19; F 4) Age: 3–6 years	Home-based, family-centred music therapy (songs, improvisation, structured music interactions), plus standard care, *n* = 12	Standard care, *n* = 11	16 sessions, scheduled weekly	Vineland SEEC, SRS-Preschool Version (parent rated), MBCDI-Words and Gestures (parent-rated) PCRI (parent-rated)	Statistical significant difference between active treatment and control in the primary outcome (Vineland SEEC-socialization). No statistical difference in the other scales	Parent not blinded to the intervention Small sample size

Boso et al. [61]	2007	Open label Duration: 52 weeks	*n* = 8 (M 7; F 1) Age: 23–38 years	Interactive music therapy (singing, piano playing, and drumming)	None	1 hour/week	CGI-Severity; CGI-Improvement, BPRS	Statistically significant improvements on the CGI-Severity; CGI-Improvement, and BPRS scale	Open label trial (no randomization, no control group) Raters not blinded Small sample size

Iseri [62]	2014	Open label Duration: 4–8 months	*n* = 10 (M 6; F 4) Age: 6–15 years	Music therapy	None	One 5-hour MT session/month	CARS, Neurohormonal responses (cortisol, adrenalin, noradrenalin, ACTH)	Decreasing scores at CARS. No statistical differences between hormone levels before and after therapy	Open label trial (no randomization, no control group) Small sample size Unclear duration Unclear compliance

Kalas [63]	2012	Crossover Duration: 3 weeks	*n* = 30 (M 28; F 2) (15 mild/moderate ASD, 15 severe ASD) Age: 4–6 years	Simple music listening	Complex music listening	Six, 10-minute individual music conditions (3 simple and 3 complex)	Responses to joint attention	Higher joint attention in the simple music condition for severe ASD. Higher joint attention in the complex music condition for mild/moderate ASD	No randomization Blinding not reported No standardized outcome measures

Lundqvist et al. [64]	2009	Randomized, crossover Duration: 10 weeks	*n* = 20 (M 13; F 7) Age: 22–57 years Diagnosis of ASD only in 10 patients; each patient had a diagnosis of mental retardation	Vibroacoustic music treatment (5 weeks)	Placebo = no treatment (5 weeks)	Two 20 min sessions per week	BPI (self-injurious behavior; stereotypical behavior; aggressive behavior) Behavior observation analysis by video recording	In ASD, vibroacoustic music statistically reduced self-injurious, behaviors. No other effect was observed	Blinding not reported Small sample size Not specific for ASD diagnosis (they included mental retardation) No accounting for potential confounders (medication, other therapies) Only one standardized measure

Schwartzberg and Silverman [65]	2013	Cluster randomized, placebo controlled (three different clusters according to the social story type) Duration: 3 weeks	*n* = 87 (no data on age or gender) Completers *n* = 30 (M 29; F 1) Age: 9–21 years	Music therapy groups (social story sung to them)	Nonmusic control groups (social story read to them)	50-min music therapy session/day per 1 week	ASSP (parent-rated 1 week before treatment and posted 1 week after) Five comprehension check questions	No significant difference between groups	Blinding not reported High drop-out rate (no information provided) Only per protocol analysis No accounting for potential confounders (medication, other therapies)

ASD, autism spectrum disorder; ASSP, Autism Social Skills Profile; BPI, Behavior Problems Inventory; BPRS, Brief Psychiatric Rating Scale; CARS, Childhood Autism Rating Scale; CGI, Clinical Global Impression; ESCS, Early Social Communication Scale; MBCDI, MacArthur-Bates Communicative Development Inventories; PCRI, Parent-Child Relationship Inventory; PDD-BI, Pervasive Developmental Disorder-Behavior Inventory; SRS, Social Responsiveness Scale; Vineland SEECS, Vineland Social Emotional Early Childhood Scales.

**Table 6:** Auditory Integration Training in ASD.

Author	Year	Type and duration of study	Sample size	Type of intervention	Comparators	Dose	Outcome measure	Findings	Comments
Bettison [66]	1996	Randomized, single blind, parallel group, placebo controlled Duration: 12 months	*n* = 80 (M 66; F 14) Age: 3–17 years	AIT according to Berard, *n* = 40	Music unmodified *n* = 40	Two 30 min sessions for 10 consecutive days	ABC, DBC, SSQ, SP (baseline, 1–3–6–12 months after intervention)	No differences between treatment and control group	Only blinding of outcome assessors Statistical analysis not optimal

Edelson et al. [67]	1999	Randomized, single blind, parallel group, placebo controlled Duration: 3 months	*n* = 19 (M 17; F 2) Age: 4–39 years	AIT according to Berard, *n* = 9	Music unmodified *n* = 10	Two 30 min sessions for 10 consecutive days	ABCa, CRS, FAPC, auditory processing tests (SCAN and SSW), electrophysiological recordings (P300 ERP) (baseline, 1-2-3 months after)	Statistical significant difference in ABCa at 3 months	Only blinding of outcome assessors Statistical analysis not optimal Small sample size

Mudford et al. [68]	2000	Randomized, single blind, crossover Duration: 14 months	*n* = 21 (M 17; F 4) Drop-out *n* = 5 Age: 5.75–13.92 years	AIT according to Berard	Similar but with nonfunctional headphones and unmodified music	Two 30 min sessions for 10 consecutive days	ABCa (baseline and every month after), direct observation of behavior (baseline and every month after), Vineland, Reynell Language Developmental Scales-III, Leiter (baseline and month 14)	No significant difference between the two groups	Small sample size High drop-out rate (low compliance) Per-protocol analysis, no intention to treat

Rimland and Edelson [69]	1995	Randomized, single blind, parallel group Duration: 3 months	*n* = 18 (M 12; F 6) Drop-out *n* = 1 Age: 4–21 years	AIT according to Berard, *n* = 9	Music unmodified, *n* = 9	Two 30 min sessions for 10 consecutive days	ABCa, FAPC, HSQ (baseline, 2 weeks, and 1-2-3 months after)	Significant improvement in ABCa and FAPC for the experimental group	Significant difference at baseline from patients in the AIT and control group Small sample size Experimenter not blinded (while parents and assessors of outcome were blinded)

Veale [70]	1993	Randomized, single blind, parallel group Duration: 3 months	*n* = 10 Age: approximately 6–10 years	AIT according to the Clark method	Music unmodified	Two 30 min sessions for 10 consecutive days	ABCa, CRS, FAPC	Significant improvement in ABCa in the experimental group	Investigator not blinded Insufficient data on baseline characteristics of patients Small sample size

Zollweg et al. [71]	1997	Randomized, double blind, parallel group Duration: 9 months	*n* = 30 Age: 7–24 years Diagnosis of ASD only in 9 patients	AIT	Music unmodified	Two 30 min sessions for 10 consecutive days	ABCa	No differences between treatment and control group	Not specific for ASD Statistical analysis carried out on different sample size (28 for ABCa, 22 for loudness discomfort, 14 for pure tone threshold)

ABC, Autism Behavior Checklist; ASD, autism spectrum disorder; CRS, Conners' Rating Scales; DBC, Developmental Behavior Checklist; FAPC, Fisher's Auditory Problems Checklist; HSQ, Hearing Sensitivity Questionnaire; SCAN, Screening Test for Auditory Processing Disorders; SP, Sensory Problem; SSQ, Sound Sensitivity Questionnaire; SSW, Staggered Spondaic Word Test.

**Table 7:** Sensory integration therapy in ASD.

Author	Year	Type and duration of study	Sample size	Type of intervention	Comparators	Dose	Outcome measure	Findings	Comments
Fazlioglu and Baran [72]	2008	Randomized, parallel group Duration: 12 weeks	*n* = 30 (M 24; F 6) Age: 7–11 years	Sensory diet consisting of brushing and joint compression followed by activities liked by the child and integrated in the daily routine (*n* = 15)	Control (*n* = 15)	24 45-min sessions (2 days a week)	Checklist developed by the investigators to quantify severity of sensory processing abnormalities	Significant improvement in treatment group	Small sample size Blinding not reported Not standardized outcome measure No sufficient information on treatment and control activities

Pfeiffer et al. [73]	2011	Randomized, single blind, parallel group Duration: 6 weeks	*n* = 37 (M 32; F 5) Drop-out *n* = 4 Age: 6–12 years Diagnosis of ASD only in 21 patients	Sensory integration according to Parham (*n* = 20)	Fine motor control group (*n* = 17)	18 45-minute sessions	SPM, SRS, GAS, and QNST-II	Significant improvement in mannerism and in GAS score in sensory group compared to fine motor activity	Small sample size High drop-out rate (almost 10%) Only per-protocol analysis Baseline characteristics different at baseline between the two groups QNTS-II not available for 30% of subjects in both groups

Reilly et al. [74]	1983	Randomized, crossover Duration: unclear, probably all sessions were provided in one day	*n* = 18 (M 15; F 3) Age: 6.2–11.7 years	Sensory integration	Fine motor activities (puzzle)	Two 30 min sessions	ASIEP	Significant difference in variety of speech and length of utterances favoring fine motor activity	Small sample size No standardized sensory integration therapy

Thompson [75]	2011	Open label Duration: unclear	*n* = 50 (M 26; F 24) Age: not reported Diagnosis of ASD only in 10 patients	Sensory integration according to Parham	None		Sustained focus based on observation	Significant improvement in sustained focus in patients with ASD	Small sample size Not specific for autism Open label trial Data collectors not blinded Statistical analysis not optimal No standardized outcome measure Insufficient baseline data

ASIEP, Autism Screening Instrument for Educational Planning; GAS, Goal Attainment Scale; QNST-II, Quick Neurological Screening Test-II; SPM, Sensory Processing Measure; SRS, Social Responsiveness Scale.

**Table 8:** Art-related therapies in ASD.

Author	Year	Type and duration of study	Sample size	Type of intervention	Comparators	Dose	Outcome measure	Findings	Comments
Corbett et al. [76]	2011	Open label Duration: 3 months	*n* = 8 (M 7; F 1) Age: 6 to 17 years	SENSE (Social Emotional NeuroScience Endocrinology) Theatre	None	38 rehearsals (2 h each) and six performance dates. Rehearsals were initially 1 day per week and then 3 or 4 days per week	NEPSY Memory for Faces, Affect Recognition and Theory of Mind, SRS, SSP, ABAS, SSS, salivary cortisol and oxytocin level	No differences in OT level or parent report measures. Improvement in social perception and Theory of Mind skills	Open label design Small sample size Blinding of assessors not reported

Mateos-Moreno and Atencia-Doña [77]	2013	Open label Duration: 18 weeks	*n* = 8 (M 7; F 1) Age: not reported (mean 25 years)	Combined dance/movement and music therapy	Behavioral and pharmacological treatment only	36 sessions of combined MT and DMT therapy, 1 hour, 2 days/week	ECA-R	Positive evolution towards a diminution of disorder scores in both control and experimental groups.	Open label design Small sample size Blinding of assessors not reported

ABAS, Adaptive Behavior Assessment System; ASD, autism spectrum disorder; ECA-R, Revised Clinical Scale for the Evaluation of Autistic Behavior; SRS, Social Responsiveness Scale, SSP, Short Sensory Profile, and SSS, Stress Survey Schedule for Persons with Autism and Other Developmental Delays.

**Table 9:** Acupuncture in ASD.

Author	Year	Type and duration of study	Sample size	Type of intervention	Comparators	Dose	Outcome measure	Findings	Comments
Allam et al. [78]	2008	Randomized single blind parallel group Duration: 9 months	*n* = 20 (M 12; F 8) Age: 4–7 years	Acupuncture (scalp) plus language therapy *n* = 10	Language therapy *n* = 10	Twice weekly	Arabic language test	Significant improvement in the acupuncture group	No placebo condition No detailed baseline characteristics Small sample size

Chan et al. [79]	2009	Randomized parallel group Duration: 6 weeks	*n* = 32 (M 26; F 6) Age: 4–7 years	Seven-needle star stimulation plus conventional education therapy *n* = 16	Conventional education therapy *n* = 16	One 5–10 min session per day, 5 days per week	Parent's rating questionnaire, Quantitative EEG	Significant improvement in the acupuncture group	No blinding No placebo condition No detailed baseline characteristics Small sample size No standardized outcome measures

Wong and Chen [80]	2010	Randomized double blind placebo controlled parallel group Duration: 4 weeks	*n* = 55 (M 47; F 8) Age: 3–18 years	Acupuncture (electro) plus convention educational therapy *n* = 31 Drop-out *n* = 1	Sham electroacupuncture plus convention educational therapy *n* = 28 Drop-out *n* = 3	30 min, 3 times weekly	PEDI, Leiter-R, CGI, ABC, RFRLS, RDLS, and WeeFIM	Significant improvement	Multicomponent intervention and conventional intervention vary from child to child No power calculation

Wong [81]	2010	Randomized double blind placebo controlled parallel group Duration: 8 weeks	*n* = 50 (M 44; F 6) Age: 3–11 years	Acupuncture (tongue) plus convention educational therapy *n* = 25	Sham acupuncture plus conventional educational therapy *n* = 25	One less than 15 sec session for 5 days per week	Griffiths mental developmental scale, RFRLS, RDLS, SPT, and WeeFIM	Significant improvement	Multicomponent intervention and conventional intervention vary from child to child No power calculation

ABC, Aberrant Behavioral Checklist; ASD, autism spectrum disorder; CGI, Clinical Global Impression; PEDI, Pediatric Evaluation Development Inventory; RDLS, Reynell Developmental Language Scale; RFRLS, Ritvo-Freeman Real Life Rating Scale; SPT, Symbolic Play Test; WeeFIM, Functional Independence Measure for children.

**Table 10:** Massage in ASD.

Author	Year	Type and duration of study	Sample size	Type of intervention	Comparators	Dose	Outcome measure	Findings	Comments
Escalona et al. [82]	2001	Randomized parallel group Duration: 4 weeks	*n* = 20 (M 12; F 8) Age: 3–6 years	Massage (simple touch)	Reading stories	15 min/day	Conners Teacher and Parent scales, sleep behavior	Improvement	No blinding No placebo condition

Silva et al. [83]	2007	Randomized parallel group Duration: 5 months	*n* = 15 (M 13; F 2) Age: >6 years Children stratified according to cognitive level	Massage (Qigong) plus special education program *n* = 8	Special education program *n* = 7	15 min/day	SP, Vineland, ABC	Improvement in all scale apart from ABC and Vineland language and motor abilities	Blinding not reported No placebo condition Special education program may vary between children Statistical analysis performed on 20 children (they included children in the control group who were switched to active treatment)

Silva et al. [84]	2009	Randomized, single blind, parallel group Duration: 5 months	*n* = 46 (M 37; F 9) Age: 3–6 years	Massage (Qigong) plus special education program *n* = 25	Waitlist *n* = 21	15 min/day	PDDBI (teacher and parent rated), ABC, SSC	Significant improvement in teacher-rated PDDBI	Teacher appears to be blinded but no detailed information No information on waitlist are provided No information on additional treatments No sample size calculation

Piravej et al. [85]	2009	Randomized, single blind, parallel group Duration: 8 weeks	*n* = 60 (M 49; F 11) Age: 3–6 years	Massage (Thai) plus sensory integration *n* = 30	Sensory integration *n* = 30	2 h/week	Conners' Rating Scales, sleep behavior	Significant improvement in parent-rated conduct problems and anxiety	No placebo condition Multicomponent intervention No improvement in teacher-rated scales (teachers were blinded while parents not)

ABC, Aberrant Behavioral Checklist; ASD, autism spectrum disorder; PDDBI, Pervasive Developmental Disorders Behavior Inventory; SP, Sensory Profile; SSC, Sense and Self-Regulation Checklist.

**Table 11:** Yoga in ASD.

Author	Year	Type and duration of study	Sample size	Type of intervention	Comparators	Dose	Outcome measure	Findings	Comments
Rosenblatt et al. [86]	2011	Open label Duration 8 weeks	*n* = 24 (M 22; F 2) Drop-out *n* = 9 (initially enrolled 33) Age: 3–18 years	Yoga (plus dance plus music)	None	45 min for 8 sessions	BASC, ABC	Improvement, but no change in ABC	Open label design Few baseline characteristics of study participants High drop-out rate

Koenig et al. [87]	2012	Open label pretest-posttest control group design Duration 16 weeks	*n* = 46 (M 37; F 9) Drop-out *n* = 3 (originally recruited *n* = 49) Age: 5–12 years	Yoga *n* = 25	School normal activities *n* = 24	15–20 min/day	ABC, Vineland	Improvement with moderate effect size in the experimental group	Open label design Statistical analysis not optimal Blinding not reported No placebo group

Chan et al. [88]	2013	Randomized, parallel group Duration 4 weeks	*n* = 46 (gender not reported for the all sample) Age: 6–17 years	Mind-body exercise (Nei Yang Gong) *n* = 23 Drop-out *n* = 3	Progressive Muscle Relaxation *n* = 23 Drop-out *n* = 3	Twice per week	Tower of London Test, CCTT, Five Point Test, ATEC, event-related EEG assessment	Significant improvement in the experimental group in self-control	Blinding not reported No placebo condition Only per protocol analysis

ABC, Aberrant Behavioral Checklist; ASD, autism spectrum disorder; ATEC, Autism Treatment Evaluation Checklist; BASC, Behavioral Assessment System for Children; CCTT, Children's Color Trails Test.

**Table 12:** Pet therapy in ASD.

Author	Year	Type and duration of study	Sample size	Type of intervention	Comparators	Dose	Outcome measure	Findings	Comments
Kern et al. [89]	2011	Open label Duration: 12 months	*n* = 24 (M 18; F 6) Age: 3–12 years	Horse riding	Waiting list	60 min lesson once a week	CARS, the Timberlawn Parent-Child Interaction Scale, SP	Improvement in CARS	Open label design No blinding Waiting list lasts for three months Unclear drop-out rate

O'Haire et al. [90]	2014	Open label Duration: 8 weeks	*n* = 64 (M 50; F 14) Age: 5–12 years	Pet therapy with Guinea pig *n* = 27	Waiting list *n* = 37	Two 20-minute sessions weekly	PDDBI, SSRS	Improvement in all outcome measures	Open label design No blinding

ASD, autism spectrum disorder; CARS, Childhood Autism Rating Scale; PDDBI, Pervasive Developmental Disorder-Behavior Inventory; SP, Sensory Profile; SSRS, Social Skills Rating System.

**Table 13:** Chiropractic care in ASD.

Author	Year	Type and duration of study	Sample size	Type of intervention	Comparators	Outcome measure	Findings	Comments
Aguilar et al. [91]	2000	Open label Duration: 9 months	*n* = 26	Chiropractic care	None	Modified Autism Checklist CARS	Improvement	No control group Open label design Improvement potentially due to regression to the mean or normal development

Khorshid et al. [92]	2006	Randomized, parallel group Duration: 3–5 months	*n* = 14 (M 13; F 1) Age: 4–16 years	Atlas Orthogonal Upper Cervical SMT *n* = 7	Full spine SMT *n* = 7	ATEC	Improvement (more in AO)	Small sample size No control group Blinding not reported

ASD, autism spectrum disorder; ATEC, Autism Treatment Evaluation Checklist; CARS, Childhood Autism Rating Scale; SMT, Spinal Manipulative Therapy.
